# Hybrid closed-loop therapy in adults with type 1 diabetes in England: Long-term outcomes from a real-world observational study

**DOI:** 10.1089/dia.2025.0165

**Published:** 2025-05-30

**Authors:** Alexandros L. Liarakos, Thomas S.J. Crabtree, Tomás P. Griffin, Sufyan Hussain, Geraldine Gallen, Jackie Elliott, Niall Furlong, Parth Narendran, Hood Thabit, Lalantha Leelarathna, Mark L. Evans, Christopher Philbey, Iain Cranston, Shafie Kamaruddin, Zin Zin Htike, Lynn Sawyer, Louise Curtis, Jesina Kirby, Isy Douek, Ali J. Chakera, Simon Saunders, Alex Bickerton, Zosanglura Bawlchhim, Clare Soar, Claire Wadham, Claire Williams, Mindy Levitt, Philip Weston, Partha Kar, Robert E.J. Ryder, Alistair Lumb, Pratik Choudhary, Emma G. Wilmot

**Affiliations:** 1 Department of Diabetes & Endocrinology, University Hospitals of Derby and Burton NHS Foundation Trust, Derby, United Kingdom; 2 University of Nottingham, Faculty of Medicine and Health Sciences, Nottingham, United Kingdom; 3 Leicester Diabetes Centre, University Hospitals of Leicester NHS Foundation Trust, Leicester, United Kingdom; 4 University of Leicester, Diabetes Research Centre, Leicester, United Kingdom; 5 Department of Diabetes and Endocrinology, Guy’s and St. Thomas’ NHS Foundation Trust, London, United Kingdom; 6 Department of Diabetes, School of Cardiovascular, Metabolic Medicine and Sciences, King’s College London, London, United Kingdom; 7 Institute of Diabetes, Endocrinology and Obesity, King’s Health Partners, London, United Kingdom; 8 Kings College Hospital NHS Trust, London, United Kingdom; 9 Department of Diabetes & Endocrinology, Northern General Hospital, Sheffield, United Kingdom; 10 University of Sheffield, Sheffield, United Kingdom; 11 Department of Diabetes & Endocrinology, St Helens and Knowsley Hospitals NHS Trust, United Kingdom; 12 Department of Diabetes & Endocrinology, Queen Elizabeth Hospital, Birmingham, United Kingdom; 13 University of Birmingham, The Institute of Immunology and Immunotherapy, Birmingham, United Kingdom; 14 Diabetes, Endocrinology and Metabolism Centre, Manchester University NHS Foundation Trust, Manchester, United Kingdom; 15 Division of Diabetes, Endocrinology and Gastroenterology, Faculty of Biology, Medicine and Health, University of Manchester, Manchester, United Kingdom; 16 Department of Metabolism, Digestion and Reproduction, Imperial College London, London, United Kingdom; 17 Wolfson Diabetes and Endocrine Clinic, Cambridge University Hospitals NHS Foundation Trust, Cambridge, United Kingdom; 18 Institute of Metabolic Science, University of Cambridge, Cambridge, United Kingdom; 19 Department of Diabetes & Endocrinology, Harrogate and District NHS Foundation Trust, Harrogate, United Kingdom; 20 Department of Diabetes & Endocrinology, Portsmouth Hospitals University NHS Trust, Portsmouth, United Kingdom; 21 Department of Diabetes & Endocrinology, County Durham and Darlington NHS Foundation Trust, Durham, United Kingdom; 22 Department of Diabetes & Endocrinology, Nottingham University Hospitals NHS Trust, Queens Medical Centre, Nottingham, United Kingdom; 23 Department of Diabetes & Endocrinology, North Bristol NHS Trust, Bristol, United Kingdom; 24 Department of Diabetes & Endocrinology, Dorset County Hospital, Dorchester, United Kingdom; 25 Department of Diabetes & Endocrinology, Worthing Hospital, Worthing, United Kingdom; 26 Department of Diabetes & Endocrinology, Musgrove Park Hospital, Taunton, United Kingdom; 27 Department of Diabetes & Endocrinology, Royal Sussex County Hospital, University Hospitals Sussex NHS Foundation Trust, Brighton, United Kingdom; 28 Department of Diabetes & Endocrinology, Mersey and West Lancashire NHS Teaching Hospitals Trust, United Kingdom; 29 Department of Diabetes & Endocrinology, Yeovil District Hospital, Yeovil, United Kingdom; 30 Cedar Centre, Department of Diabetes & Endocrinology, Royal Surrey County Hospital, Guildford, United Kingdom; 31 Department of Diabetes & Endocrinology, Nottingham City Hospital, Nottingham, United Kingdom; 32 Department of Diabetes & Endocrinology, Ipswich Hospital, Ipswich, United Kingdom; 33 Department of Diabetes & Endocrinology, County Durham and Darlington NHS Foundation Trust, Darlington, United Kingdom; 34 Department of Diabetes & Endocrinology, Princess Royal Hospital, University Hospitals Sussex NHS Foundation Trust, Haywards Heath, United Kingdom; 35 Department of Diabetes & Endocrinology, Royal Liverpool University Hospital, Liverpool, United Kingdom; 36 Department of Diabetes and Endocrinology, City Hospital, Sandwell and West Birmingham Hospitals NHS Trust, Birmingham, United Kingdom; 37 Oxford Centre for Diabetes Endocrinology and Metabolism, Oxford, United Kingdom; 38 National Institute for Health and Care Research, Oxford Biomedical Research Centre, Oxford, United Kingdom

**Keywords:** Hybrid closed loop, Automated insulin delivery, Artificial pancreas, Type 1 diabetes, Diabetes technology

## Abstract

**Objective:**

To evaluate longitudinal real-world outcomes in adults with type 1 diabetes initiating hybrid closed-loop (HCL).

**Methods:**

Adults with type 1 diabetes, managed with an insulin pump and intermittently scanned continuous glucose monitoring with an HbA1c ≥8.5% (69mmol/mol), were started on HCL between August and December 2021 as part of the National Health Service England HCL pilot. We collected outcomes including change in HbA1c, sensor glucometrics, Gold score (hypoglycemia awareness), diabetes distress score, acute event rates, and user opinion of HCL.

**Results:**

In total, 420 HCL users across 30 diabetes centers in the UK were included (median age 40 [IQR 29-50] years, 68% female, 85% White British). Over a median follow-up of 12 months (IQR 8-28) (range 6-38 months), mean adjusted HbA1c reduced by 1.4% (95% CI -1.5, -1.3; P<0.001) (16mmol/mol [95% CI -17, -14]; P<0.001). Time in range (70-180mg/dL) increased from 33.7% to 60.4% (P<0.001). The proportion of individuals achieving HbA1c≤7.5% (58mmol/mol) increased from 0 to 33.1% (P<0.001). Diabetes distress score reduced (-1.1; 95% CI -1.3, -1.0; P<0.001) and Gold score reduced (-0.4; 95% CI -0.5, -0.2; P <0.001). The percentage of individuals with impaired hypoglycemia awareness (Gold score≥4) decreased (16.6% [baseline] vs. 9.2% [follow-up]; P<0.001). Almost all participants stated HCL had a positive impact on quality of life (94.5%; 361/382). The number of hospital admissions was low.

**Conclusions:**

Long-term real-world use of HCL is associated with sustained improvements in glycemic and person-reported outcomes in adults with type 1 diabetes and above-target HbA1c levels.

## Introduction

Type 1 diabetes is a chronic condition that requires life-long insulin therapy and necessitates affected people to make multiple decisions daily. For most individuals with type 1 diabetes, making frequent decisions is difficult, impacts quality of life (QoL) and is associated with dysglycemia which can result in complications, increased hospitalization and mortality as well as high rates of diabetes-related distress and depression.^1–5^ Despite best attempts with access to diabetes specific education and continuous glucose monitoring, less than a third of people with type 1 diabetes achieve the recommended glucose outcomes^6^ while many face a significant burden in managing their condition.^1^

Randomized controlled trial (RCT) evidence has shown improvements in hemoglobin A1c (HbA1c), hypoglycemia and psychological outcomes with hybrid closed-loop (HCL) compared with usual care.^7,8^ These RCTs (7) assessing the effects of HCL in type 1 diabetes typically involve people with a lower mean baseline HbA1c compared with the levels observed in real-life clinical settings. In contrast, real-world evaluation studies capture outcomes of a given therapy in a broader population, including individuals often excluded from RCTs. Although existing real-world data have demonstrated the benefits of HCL across different countries and with different systems,^9–11^ these data do not capture HbA1c levels or patient-reported outcomes over time.

Notably, our previous 5-month real-world study^12^ evaluating HCL use in adults with type 1 diabetes in the UK showed significant improvements in HbA1c and reductions in hypoglycemia. These findings informed the National Institute for Health and Care Excellence (NICE) leading to the approval of the Technology Appraisal 943 (TA943) guidance on the use of HCL systems in type 1 diabetes in England.^13^ Nevertheless, despite these promising short-term outcomes, there remains a need for longitudinal follow-up data to assess the sustained efficacy and QoL improvements over time, which is crucial for informing long-term clinical practice and healthcare decision-making. Hence, the aim of this study was to evaluate long-term real-world clinical and user-reported outcomes associated with HCL in adults with type 1 diabetes in the United Kingdom (UK).

## Materials and Methods

### Patient recruitment and data collection

The methodology for this observational study has been previously described.^14^ Data for this study capture the real-world outcomes from individuals attending adult diabetes services with a diagnosis of type 1 diabetes managed with an insulin pump and intermittently scanned continuous glucose monitoring (CGM) (isCGM) with an HbA1c ≥8.5% (69 mmol/mol), who started HCL between August and December 2021, as part of the NHS England adult HCL pilot.^14^ The diabetes services selected for the pilot were those with the highest number of insulin pump users and, consequently, the greatest experience in diabetes technology. Anonymized clinical data were collected during routine clinical care, and clinical systems and electronic health records were reviewed. Device-derived metrics, including sensor glucometrics, were extracted from summary reports generated by commercial web-based platforms, such as LibreView, CareLink™ and Glooko. Data were submitted to a secure web-based tool within the NHS network (https://abcd.care/audit/abcd-nhs-england-nationwide-closed-loop-pilot-audit). This analysis reflects the data captured between 6 and 38 months of follow-up, from March 2022 to October 2024.

### Outcome measures

The primary outcome was change in HbA1c. Secondary outcomes included change in 14-day CGM-derived mean percentage of time spent in 70–180 mg/dL (3.9–10.0 mmol/L) time in range (TIR), at 181–250 mg/dL (10.1-13.9 mmol/L) (time above range [TAR], level 1), at >250 mg/dL (>13.9 mmol/L) (TAR, level 2), at 54–69 mg/dL (3.0-3.8 mmol/L) (time below range [TBR], level 1), at <54 mg/dL (<3.0 mmol/L) (TBR, level 2), glucose management indicator (GMI) (estimated HbA1c), and percentage co-efficient of variation. Other outcomes included event rates (hospital admissions, paramedic callouts, severe hypoglycemia requiring third party assistance). Diabetes distress was assessed using the two-item diabetes distress screening instrument (DDS2) (question 1 “feeling overwhelmed by the demands of living with diabetes”, question 2 “feeling that I am often failing with my diabetes regimen”).^15^ Gold score was used to assess hypoglycemia awareness.^16^ User opinion of HCL (question 1 “would you recommend HCL to other people with diabetes” and question 2 “what impact would you rate HCL has had on your quality of life”; a 7-point Likert scale was used: 1 = would not recommend at all, 7 = would highly recommend [for question 1]; 1 = extremely negative impact, 7 = extremely positive impact [for question 2]) was sought. Demographic data included age, gender, ethnicity, weight, body mass index (BMI), and index of multiple deprivation decile (a measure of relative deprivation at a small local area level in the UK; 1 = most deprived, 10 = least deprived). Follow-up frequency was determined by the responsible clinical team based on clinical need. Data were captured at baseline (for the 12 months prior to HCL initiation) and at follow-up (during routine clinical follow up). CGM glucometrics were assessed for the 14 days prior to HCL initiation and extracted from the relevant HCL system for the 14 days preceding follow-up.

### Ethical approval

The ABCD national audit program, which includes the ABCD NHS England nationwide closed loop pilot audit, has Caldicott Guardian Approval and has been assessed by the Confidentiality Advisory Group.^17^ The program captures anonymized and routinely available clinical data. Tests not done routinely were not needed to be performed. Hence, this study did not require specific approval by a research ethics committee.

### Statistical methods

Continuous variables were presented as mean ± standard deviation (SD) or median and interquartile range (IQR) and assessed using paired t-test or Wilcoxon signed rank test depending on normality of distribution (determined by Shapiro-Wilk test and Kolmogorov-Smirnov test). Categorical variables were expressed as numbers and percentages and assessed using Chi-squared tests. To address loss to follow-up, the analysis of each outcome of interest included only individuals who had available data at both baseline and follow-up. A two-sided p-value <0.01 was considered statistically significant to account for multiplicity. Change in HbA1c from baseline was adjusted for several key covariates determined a-priori (baseline HbA1c, age, gender, ethnicity, deprivation status, duration of diabetes, duration of pump therapy at baseline, HCL system, time in closed loop) using a multivariable linear regression model with estimated marginal means. Mann Whitney U (Wilcoxon Rank Sum) tests were used to assess changes in patterns of hospital admissions and paramedic callouts. Statistical analysis was performed on SPSS v28.0 (IBM, Chicago, IL). ALL and TSJC are the guarantors of this work and, as such, had full access to all the data in the study and take responsibility for the integrity of the data and the accuracy of the data analysis.

## Results

Baseline data were available for 488 adults, with follow-up data reported for 435 individuals, of whom 420 (96.6%) continued to use HCL across 30 centers in the UK. The median follow-up was 12 months (IQR 8-28) (range 6-38 months). Supplement 1 depicts the flow diagram for this analysis.

### Baseline characteristics

For the 420 individuals, with baseline and follow-up data, the median age was 40 years (IQR 29-50); 68% (n = 285) were female; 85% (n = 356) were White British; median diabetes duration was 21 years (IQR 15–29) and insulin pump use was 8 years (IQR 5-11). 9% (36 of 420) were from the most deprived quintile and 14% (57 of 420) were from the least deprived quintile, with a median index of multiple deprivation decile of 6 (IQR 3–8). The baseline characteristics of the cohort are shown in Table 1. The HCL systems used in our population included Medtronic 780G (47%, n = 199), Tandem Control-IQ (36%, n = 150), CamAPS FX (4%, n = 18), Medtrum HCL (4%, n = 17) and Medtronic 670G (2%, n = 8); the system was not reported for 28 (7%) people.

### Glycemic outcomes

**Figure 1:**
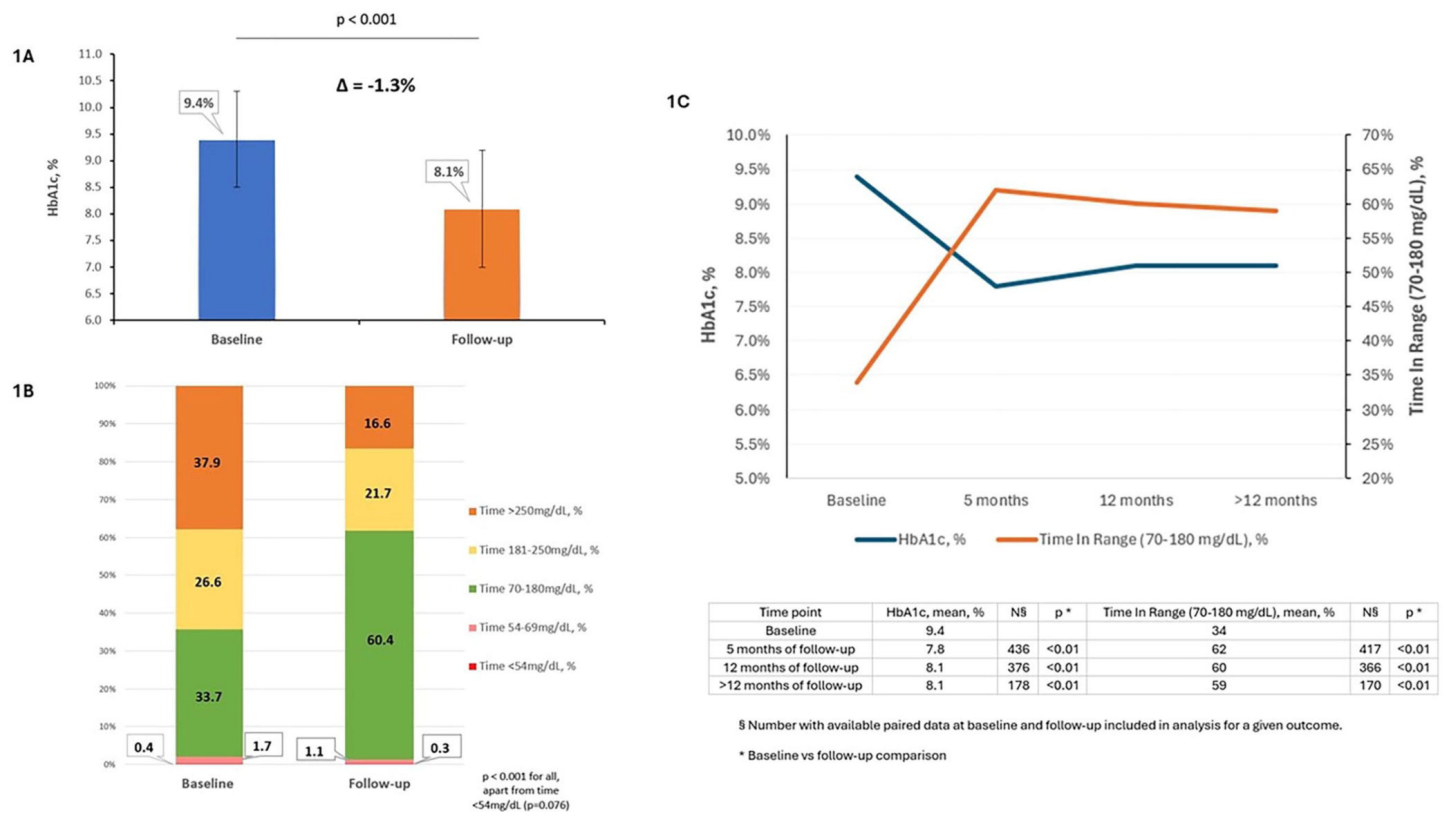
A: HbA1c (%) at baseline and follow-up (n = 376) (uncorrected change). The error bars indicate standard deviation. B: Stacked bar chart showing the time spent in glucose ranges at baseline and follow-up (uncorrected changes). Available paired baseline and follow-up data were included only. Data derived from intermittently scanned continuous glucose monitoring at baseline and real-time continuous glucose monitoring at follow-up. C: HbA1c (%) and time in range (70-180 mg/dL) (%) outcomes over time.

HbA1c reduced from 9.4 ± 0.9% (79 ± 10 mmol/mol) at baseline to 8.1 ± 1.1% (65 ± 12 mmol/mol) at follow-up, a mean unadjusted reduction of 1.3% (14 mmol/mol) [95% CI -1.4, -1.2; P < 0.001), over a median follow-up of 12 months (IQR 8-28) (range 6-38 months) (Figure 1A). Using a multivariable linear regression model to correct for key confounders, the mean adjusted HbA1c reduction was 1.4% (95% CI -1.5, -1.3; P < 0.001) (16 mmol/mol [95% CI -17, -14]; P < 0.001).

The median time spent in closed-loop mode was 95% (IQR 90–98). The GMI reduced from 8.7% (72 mmol/mol) at baseline to 7.4% (57 mmol/mol) at follow-up (−1.3% [95% CI −1.4, −1.2]; P < 0.001) (−15 mmol/mol [95% CI −16, −13]; P < 0.001). Mean TIR (70–180 mg/dL, 3.9–10.0 mmol/L) increased from 33.7% to 60.4%, an increase of 26.7% (95% CI 25.0, 28.3; P < 0.001). Mean TAR level 1 hyperglycemia (181–250 mg/dL, 10.1-13.9 mmol/L) reduced from 26.6% to 21.7%, a decrease of 4.9% (P <0.001), and mean TAR level 2 hyperglycemia (>250 mg/dL, >13.9 mmol/L) reduced from 37.9% to 16.6%, a decrease of 21.3% (P <0.001). Mean TBR (<70 mg/dL, <3.9 mmol/L) decreased from 2.1% to 1.4% (P <0.001) with level 1 hypoglycemia (54–69 mg/dL, 3.0-3.8 mmol/L) decreasing by 0.6% (P <0.001) and level 2 hypoglycemia (<54 mg/dL, <3.0 mmol/L) reducing by 0.1% (P = 0.076). The mean percentage co-efficient of variation (CV) decreased from 37.8% at baseline to 35.0% at follow-up (−2.8% [95% CI −3.9, −1.8]; P < 0.001). Among individuals with paired baseline and follow-up CV data, the proportion of those meeting the target of CV ≤36% increased from 38.2% (115 of 301) at baseline to 56.8% (171 of 301) at follow-up (P = 0.01). The changes in CGM-derived glucose metrics are depicted in Figure 1B. The paired HbA1c and CGM-derived glucose metric outcomes are shown in Table 2 and Supplement 2.

Importantly, the change in HbA1c was not associated with gender, ethnicity, deprivation status or the HCL system used (P >0.05 for all). The change in HbA1c was similar between individuals (n = 173) with <12-month follow-up data and those (n = 203) with ≥12-month follow-up (P = 0.6). The HbA1c and TIR outcomes over time are depicted in Figure 1C.

**Figure 2:**
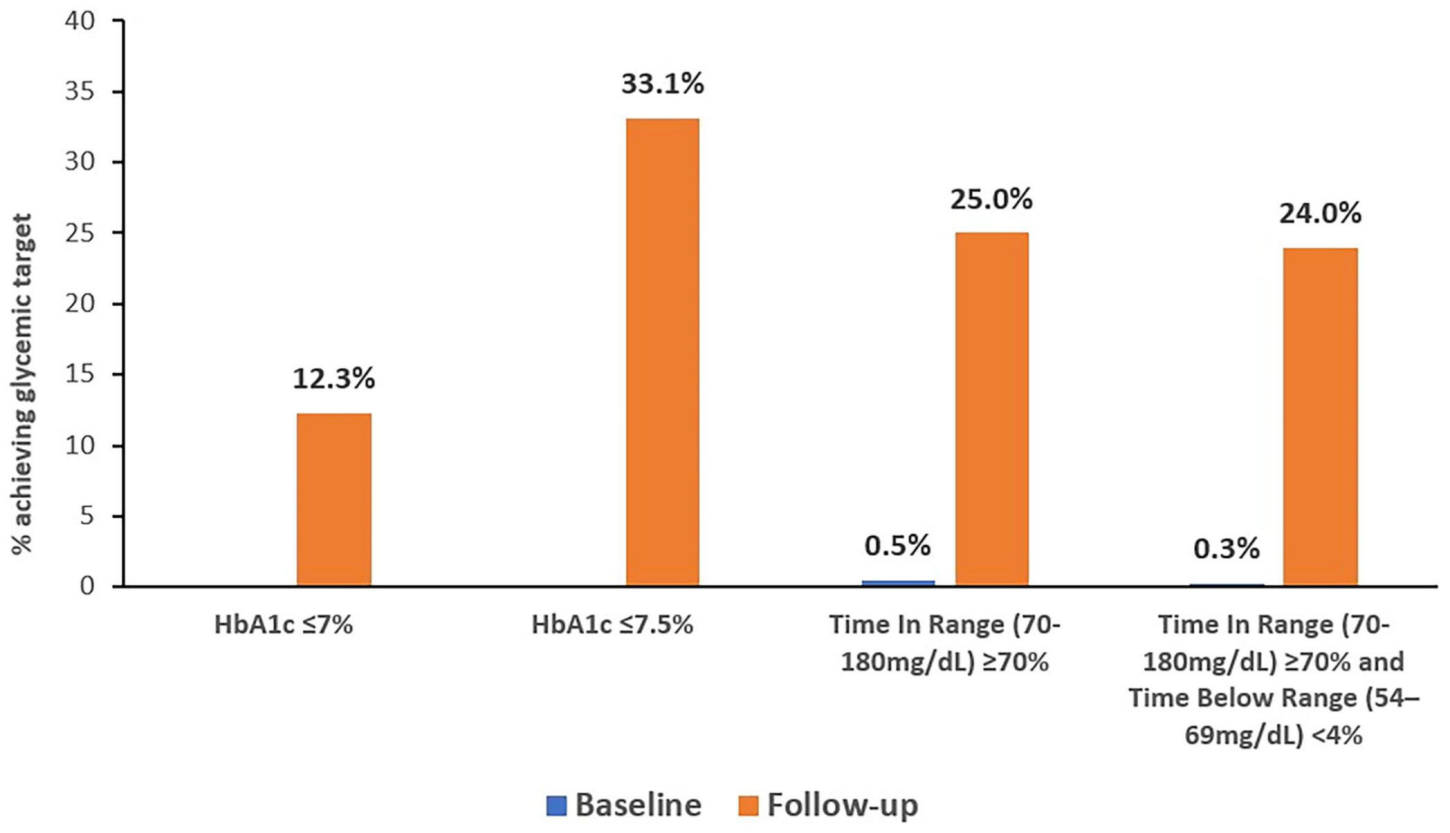
Proportion of individuals achieving internationally recommended glycemic targets at baseline and follow-up.

As per entry criteria, no people had an HbA1c ≤7.5% (58 mmol/mol) at baseline, while 33.1% (126 of 381) of HCL users met this HbA1c target at follow-up, and 12.3% (47 of 381) achieved HbA1c ≤7% (53 mmol/mol). The proportion of the population achieving the target of TIR ≥70% increased from 0.5% at baseline to 25.0% (100 of 400) at follow-up (P < 0.001) while the proportion achieving the composite target of ≥70% TIR and <4% TBR increased from 0.3% to 24% (96 of 400) at follow-up (P < 0.001) (Figure 2). Among individuals with paired baseline and follow-up HbA1c data, 84.6% (318 of 376) had an HbA1c reduction ≥0.5%, with 69.4% (262 of 376) and 23.1% (87 of 376) achieving reductions of ≥1.0% and ≥2.0%, respectively. A small number of people (23 of 376 [6.1%]) had an increase in HbA1c. This was associated with higher baseline GMI (OR 1.05; P = 0.009), an elevated baseline Gold score (OR 1.50; P = 0.002) and impaired awareness of hypoglycemia (Gold score ≥4) (OR 3.56; P = 0.008) as well as lower time spent in closed-loop mode (OR 0.96; P <0.001).

### Body weight, body mass index, and total daily dose of insulin

Body weight increased by 2.2 kg (81.9 ± 18.9 vs 84.1 ± 20.3 kg; 95% CI 1.4, 2.9; P < 0.001; n = 265) and body mass index increased by 0.7 kg/m^2^ (28.9 vs 29.6 kg/m^2^; 95% CI 0.5, 1.0; P < 0.001; n = 261) compared with baseline. Total daily dose of insulin increased from 46.6 ± 18.5 units at baseline to 50.7 ± 19.6 units at follow-up, an increase of 4.1 units (95% CI 2.3, 6.0; P < 0.001; n = 243).

### Patient-reported outcomes

DDS2 score reduced from 3.3 ± 1.2 at baseline to 2.2 ± 1.0 at follow-up (-1.1; 95% CI -1.3, -1.0; P < 0.001). The proportion of people with high diabetes distress (DDS2 ≥3) decreased from 67.4% at baseline to 23.1% at follow-up (P < 0.001). Gold score reduced by 0.4 (2.2 ± 1.4 vs 1.8 ± 1.2; 95% CI -0.5, -0.2; P < 0.001). Impaired awareness of hypoglycemia (Gold score ≥4) was reported in 16.6% of the population at baseline compared with 9.2% at follow-up (P < 0.001). The results of DDS2 and Gold score are summarized in Table 2. Regarding user satisfaction, 94.5% (361/382) of the respondents stated that HCL therapy has had a positive impact on their QoL, and 96.6% (370/383) of the respondents would recommend HCL to other people living with diabetes.

### Acute and adverse events

Reported hospital admissions related to hyperglycemia/diabetic ketoacidosis or severe hypoglycemia, paramedic callouts (without resulting in hospital admission) and episodes of severe hypoglycemia (without leading to paramedic callouts or hospitalization) were low in this cohort (Supplement 3). A total of 36 adverse events were reported. The majority of these events were associated with sensor failures, inaccuracies and skin reactions (19 of 36; 52.8%) followed by infusion set and/or pump failures (8 of 36; 22.2%). Other reported adverse events included connectivity issues (4 of 36; 11.1%), weight gain (3 of 36; 8.3%) and worsening of retinopathy (2 of 36; 5.6%). Both cases of worsening retinopathy occurred in individuals with a high baseline HbA1c (9.3–9.4%), with one experiencing a 1.4% reduction in HbA1c at follow-up. 15/435 (3.4%) discontinued HCL therapy after 10 months (IQR 8.5-12) (no adequate data on the reasons for discontinuation were reported).

## Discussion

This real-world evaluation of long-term (up to 38 months) use of HCL in adults with type 1 diabetes demonstrates sustainable improvements in glucose outcomes, hypoglycemia awareness, diabetes-related distress and user satisfaction. Hospital admissions due to hypoglycemia, hyperglycemia or DKA were small in number and paramedic use did not increase.

Our previous analysis using the 5-month real-world data of the NHS England HCL pilot in adults demonstrated a reduction in the mean adjusted HbA1c of 1.7% and an increase in TIR of 27.6%.^12^ The current analysis, which includes those individuals with follow-up data ranging from 6 to 38 months, showed that these significant improvements in glucose levels are sustained over time. Similarly, the NHS England Closed Loop Study in Children and Young People demonstrated significant improvements in glycemia over 12 months.^18^ Taken together, our analysis complements the long-term outcomes observed in children and young people with type 1 diabetes in the real world, suggesting the sustained glycemic benefits of HCL systems across all age groups. To date, our analysis is the longest real-world study assessing the effects of multiple different commercially available HCL systems in type 1 diabetes.

The reduction in HbA1c of 1.4% (16 mmol/mol) is greater than the one observed in RCTs and other real-world studies, which showed an HbA1c decrease of 0.4% between baseline and follow-up.^7,19^ Also, our analysis demonstrated an increase in TIR of 26.7% with HCL, which is higher than the changes reported in other studies.^7,19^ These observations are likely related to the criteria set out by the NHS England HCL pilot, which was targeted at those who were facing challenges to achieve target glycemic control despite a high level of care with insulin pump and CGM. Other studies have often included participants with tighter baseline glycemic control.^20–23^ A similar sustained decrease in HbA1c of around 1.5% was reported after 12 months of HCL use in the ADAPT RCT,^24^ which recruited adult participants on multiple daily insulin injections and isCGM with similar HbA1c levels (mean baseline HbA1c of 9.0% [75 mmol/mol]), compared with our population. Similarly, a recent study^25^ investigating the effectiveness of HCL in a pediatric population found that individuals with higher baseline HbA1c experienced the greatest improvement in glycemia.

Importantly, our analysis also found that the change in HbA1c was not associated with gender, ethnicity or deprivation status. This suggests that, in real-world settings, all users benefit equally from HCL therapy. These findings highlight the importance of ensuring that anyone meeting the eligibility criteria has equitable access to these systems and their potential benefits.

Our analysis showed lower TIR at follow-up, compared with other HCL studies in real-life clinical settings,^22,26,27^ which is explained by the much lower baseline TIR (33.7%) of our population. Our results are similar to those in the RCT by Schoelwer et al.,^28^ which showed that individuals with type 1 diabetes with the lowest TIR at baseline (approximately 39%) achieved 54% TIR, compared with 60% in our real-world study. Nevertheless, a significant increase in TIR of 26.7% was observed in our analysis, which was accompanied by substantial reductions in TAR without increased rates of hypoglycemia (TBR <2% at follow-up). Future studies incorporating more granular data on user behaviors (e.g. number of manual boluses, number of missed boluses) and system settings (e.g. percent autocorrections) could help identify modifiable factors that further optimize HCL performance, particularly in real-world settings.

A previous study of HCL in those with impaired awareness of hypoglycemia showed reductions in TBR but no clinically significant change in self-reported awareness.^28^ This real-world finding of restoration in self-reported awareness with HCL is novel in our analysis and needs to be confirmed in other real-world studies and RCTs. This finding could be associated with the change from isCGM to real-time CGM and the use of more advanced alarms, as previously reported.^29^

The proportion of individuals achieving the recommended target of HbA1c ≤7.5% (58 mmol/mol) in this real-world analysis increased from 0 to 33.1% at follow-up. This figure is comparable with the percentage of adults with type 1 diabetes in England and Wales achieving this target within the National Diabetes Audit in 2021-2022.^30^ The combined target of ≥70% TIR and <4% TBR (level 1 hypoglycemia) was achieved by 24% (96 of 400) of the population at follow-up. Achieving the recommended glycemic targets could be improved further in the future by optimizing the training and education provided to HCL users and healthcare professionals along with further developments in closed-loop technologies.

The initial 5-month data of HCL use showed an increase in body weight of 1.9 kg.^12^ The current analysis showed that long-term HCL use was associated with a small increase in body weight of 2.2 kg, compared with baseline. Weight gain was associated with baseline HbA1c (OR 1.05, P = 0.002) but was not associated with the change in HbA1c (P = 0.14) or baseline weight (OR 0.99, P = 0.04). Similar results of weight gain were observed in other real-world studies and RCTs.^31,32^ The weight gain could be explained by potentially increased dietary freedom after HCL initiation (e.g. more freedom to snack), but detailed mechanisms need to be explored further. Nevertheless, our analysis suggests that this initial weight gain reaches a plateau over time. The frequency of nutritional support in our population was determined by the responsible clinical teams based solely on clinical need, with potential regional variations in dietary counseling practices across the country. Addressing the possibility of initial weight gain may be helpful in counselling people with type 1 diabetes prior to starting HCL, as this may be a barrier for some individuals. While further research is required, the suggestion that this initial weight gain may plateau is helpful in guiding such discussions.

User satisfaction was high in our cohort. The majority of the respondents stated that HCL has had a positive impact on their QoL and would recommend HCL to other people living with diabetes. Diabetes-related distress also significantly improved. Similar results have been reported in other studies assessing the effects of HCL.^23,33–35^ These observations are likely to be multifactorial and explained by the substantial improvements in high and low glucose levels, which have been associated with worsening sleep, mood, cognition and daily functioning.^35–37^

Despite the high overall user satisfaction, it should be noted that a small proportion (3.4%) of individuals would not recommend HCL to other people living with diabetes. This highlights the need for further research exploring what success of HCL therapy means to people living with type 1 diabetes, what further support is needed when individuals with type 1 diabetes use HCL technologies and how industry can improve the options available to better suit individual needs. Also, future research should aim to capture the factors driving the choices of people with type 1 diabetes regarding HCL systems. As the performance and usability of these systems continue to improve in the future, this will likely impact the future experiences of HCL users and the related person-reported outcomes.

Our analysis indicated that long-term use of HCL systems in adults with significantly elevated baseline HbA1c is not associated with increased rates of hospital admissions related to severe hypoglycemia, hyperglycemia or DKA in real-life clinical settings. These results support the safety profile of HCL systems as already shown in RCTs^38^ and other real-world studies.^39,40^

The main strength of this study is the long-term follow-up period (up to 38 months), and the selection of people who were struggling to achieve targets despite a high level of care. This enabled the evaluation of the sustainability and durability of glycemic and patient-reported outcomes observed beyond the initial treatment period. To the best of our knowledge, there are no other studies assessing the real-world effects of multiple different commercially available HCL systems with longer follow-up than our analysis. Another key strength is the real-world clinical setting which allowed for the data collection from adults living with type 1 diabetes managed with several HCL systems in routine clinical practice across multiple diabetes centers in the UK. The real-world nature of our analysis provides observations which are more representative of the usual care for HCL users, irrespective of the system used, without restrictive inclusion and exclusion criteria commonly used in RCTs (e.g. exclusion of populations with high HbA1c levels) and without the additional intensive support that is offered in research environments.

The study should be interpreted within the context of its limitations. The inherent limitation of its retrospective nature along with lack of control group and recruitment of participants from diabetes centers with the greatest experience in diabetes technology can introduce the risk for selection bias. Other limitations include the possibility of unmeasured confounders related to data collection through audit and associated underreporting of adverse events. Missing data, loss of follow up, including lack of information about HCL continuation or discontinuation during follow-up and lack of information about follow-up frequency of the patients are some other limitations. Also, we acknowledge that our population was predominantly female and of White British ethnicity, which limits the generalizability of the results to other groups. The findings reported are less generalizable to individuals with lower baseline HbA1c levels, however, HCL use is supported in these people by existing RCT and real-world evidence. The analysis of CGM-derived metrics compared the data from isCGM (FreeStyle Libre) at baseline with real-time CGM (the type of device varied depending on the system used) at follow-up. As such, the data capture and accuracy may differ among these devices. Lastly, the expertise of the diabetes teams (HCL users were onboarded in centers with recognized pump expertise and high levels of pre-existing pump use) may have partially affected the results observed. Further real-world research is needed to assess whether similar outcomes could be achieved in centers with less experience.

## Conclusions

In conclusion, our analysis indicated that long-term real-world use of HCL was associated with significant and sustained improvements in glycemic and person-reported outcomes in adults with type 1 diabetes managed with insulin pump and isCGM, with HbA1c levels above target. These benefits were achieved without increased rates of hospital admissions, severe hypoglycemia, DKA, or paramedic use.

## Supplementary Material

Supplementary Material

## Figures and Tables

**Table 1:** Baseline characteristics of total population (N = 420)

Characteristic	Value	N
Age, years, median (IQR)	40 (29-50)	357
Gender, number (percentage)		420
Female	285 (68)	
Male	135 (32)	
Ethnicity, number (percentage)		420
White, British	356 (85)	
Asian	11 (3)	
Mixed	9 (2)	
White, other	9 (2)	
Black	3 (0.5)	
Other	2 (0.5)	
Unknown	30 (7)	
Index of multiple deprivation (IMD) decile, median (IQR)	6(3-8)	420
IMD quintiles, number (percentage)		
1st quintile (the most deprived 20% of areas; IMD 1-2)	36 (8.6)	
2nd quintile (IMD 3-4)	49 (11.7)	
3rd quintile (IMD 5-6)	39 (9.3)	
4th quintile (IMD 7-8)	53 (12.6)	
5^th^ quintile (the least deprived 20% of areas; IMD 9-10)	57 (13.5)	
Not recorded	186 (44.3)	
Diabetes duration, years, median (IQR)	21 (15-29)	358
Pump duration, years, median (IQR)	8 (5-11)	324
Weight, kg, mean ± SD	82 ± 18	343
BMI, kg/m2, mean ± SD	29 ± 6	318
HbA1c, mean ± SD		414
%	9.4 ± 0.9	
mmol/mol	79 ± 10	
Total daily insulin dose, units, mean ± SD	50 ± 24	369
CGM metrics, mean ± SD		
TAR, level 2 (>250 mg/dL, >13.9 mmol/L), %	38 ± 19	373
TAR, level 1 (181–250 mg/dL, 10.1–13.9 mmol/L), %	26 ± 11	363
TIR (70-180 mg/dL, 3.9–10.0 mmol/L), %	34 ± 14	377
TBR, level 1 (54–69 mg/dL, 3.0–3.8 mmol/L), %	2 ± 2	375
TBR, level 2 (<54 mg/dL, <3.0 mmol/L), %	0 ± 1	367
GMI, %	8.7± 1.0	263
GMI, mmol/mol	72 ± 11	
Coefficient of variation, %	38 ± 7	336
Scans per day at baseline, number, mean ± SD	7 ± 5	349
Closed-loop system, number (percentage)		420
Medtronic 780G	199 (47)	
Tandem Control-IQ	150 (36)	
CamAPS FX	18 (4)	
Medtrum	17 (4)	
Medtronic 670G	8 (2)	
Not recorded	28 (7)	

BMI: body mass index; CGM: continuous glucose monitoring; GMI: glucose management indicator; HbA1c: hemoglobin A1c; IQR: interquartile range; SD: standard deviation; TAR: time above range; TBR: time below range; TIR: time in range.

**Table 2:** Glycemic and patient reported outcomes at baseline and follow-up

	N§	Baseline	Follow-up	Change (95% CI)	p
HbA1c, mean ± SD					
%	376	9.4 ± 0.9	8.1 ± 1.1	-1.3 (-1.4, -1.2)	< 0.001
mmol/mol	376	79 ± 10	65 ± 12	-14 (-16, -13)	< 0.001
CGM metrics#, mean ± SD					
TAR, level 2 (>250 mg/dL), %	359	37.9 ± 19.2	16.6 ± 12.9	-21.3 (-23.3, -19.2)	< 0.001
TAR, level 1 (181-250 mg/dL), %	345	26.6 ± 10.6	21.7 ± 10.6	-4.9 (-6.5, -3.3)	< 0.001
TIR (70-180 mg/dL), %	366	33.7 ± 14.2	60.4 ± 13.4	26.7 (25.0, 28.3)	< 0.001
TBR, level 1 (54-69 mg/dL), %	363	1.7 ± 2.3	1.1 ± 1.3	-0.6 (-0.9, -0.4)	< 0.001
TBR, level 2 (<54 mg/dL), %	354	0.4 ±1.1	0.3 ± 0.6	-0.1 (-0.2, -0.02)	0.076
GMI, mmol/mol	242	72 ± 10	57 ± 6	-15 (−16, −13)	< 0.001
Coefficient of variation, %	301	37.8 ± 7.2	35.0 ± 7.5	−2.8 (−3.9, −1.8)	< 0.001
Patient-reported outcomes					
Diabetes distress scale score, mean ± SD	347	3.3±1.2	2.2 ± 1.0	-1.1 (-1.3, -1.0)	< 0.001
High diabetes distress (DDS2 score ≥3), % (n)	347	67.4 (234)	23.1 (80)	-44.3 (-154)	< 0.001
Gold score, mean ± SD	349	2.2 ± 1.4	1.8 ±1.2	-0.4 (-0.5, -0.2)	< 0.001
Impaired awareness of hypoglycemia (Gold score ≥4), % (n)	349	16.6 (58)	9.2 (32)	-7.4 (-26)	< 0.001

CGM: continuous glucose monitoring; CI: confidence interval; GMI: glucose management indicator; HbA1c: hemoglobin A1c; SD: standard deviation; TAR: time above range; TBR: time below range; TIR: time in range. The data in the table reflect on uncorrected changes.

§ Number with available paired baseline and follow-up data included in analysis for a given outcome; total cohort = 420.

ǂ Data derived from isCGM at baseline and real-time CGM at follow-up.

## Data Availability

The datasets generated during and/or analyzed in the current study are available from the corresponding author upon reasonable request.
