# Supplementary material for: Hybrid closed-loop therapy in adults with type 1 diabetes in England: Long-term outcomes from a real-world observational study
Source: Diabetes Technol Ther. Author manuscript; Available in PMC 2026 Jan 30. (PMC7618701; doi:10.1089/dia.2025.0165)
Supplement: Supplementary Material [file EMS211995-supplement-Supplementary_Material.pdf]

Supplement 1. Consort flow diagram for inclusion in this study

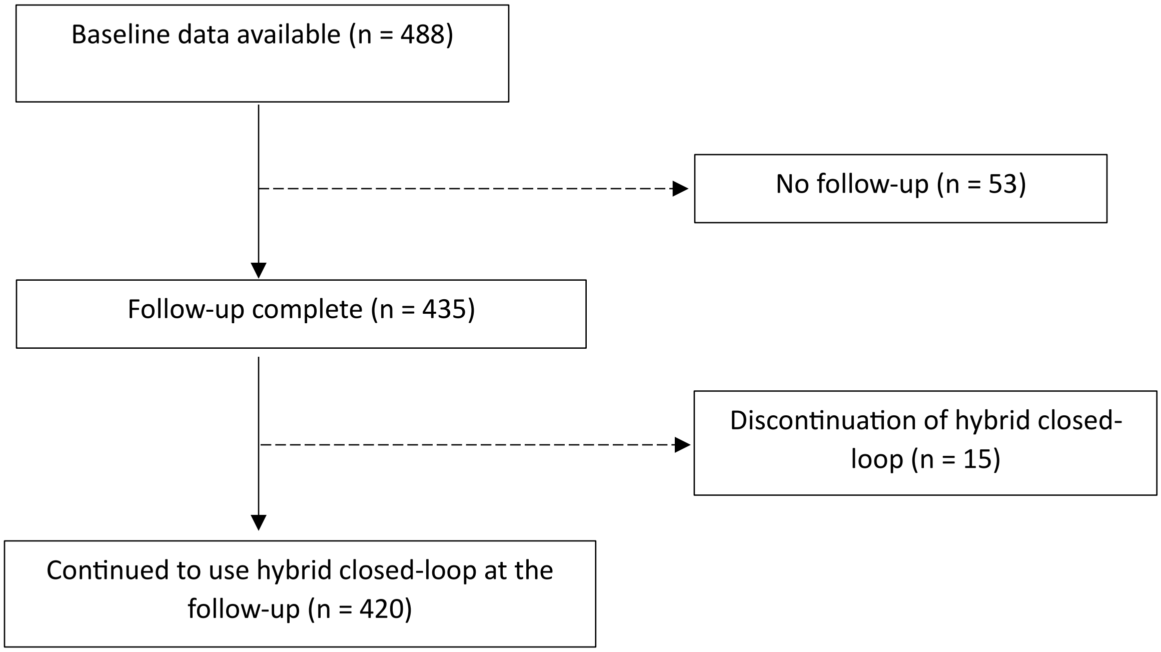

Supplement 2: Change in glycemic outcomes between baseline and follow-up, stratified by center (masked)

Center (masked)	N§	IMD decile, median (IQR)	Change in HbA1c (%) from baseline	Change in TIR (%) from baseline	Change in TAR (%) from baseline	Change in TBR (%) from baseline	Change in GMI (mmol/mol) from baseline
1	20	ND	-1.6 ± 1.2	31 ± 17	-32 ± 17	0.4 ± 2	-15 ± 10
2	2	ND	-0.9 ± 0.4	46	-36	-10	ND
3	17	8 (6-9)	-0.6 ± 1.3	34 ± 15	-32 ± 16	-1 ± 3	-17 ± 10
4	4	6 (4-7)	-1.3 ± 1.7	32 ± 19	-30 ± 19	-2 ± 2	-17 ± 8
5	54	5 (3-8)	-1.1 ± 0.8	27 ± 12	-26 ± 12	-2 ± 3	-15 ± 11
6	1	ND	-2.8	50	-52	2	-25
7	18	8 (6-10)	-1.0 ± 0.7	26 ± 13	-25 ± 14	-1 ± 2	-15 ± 8
8	5	6 (6-8)	-1.5 ± 0.6	26 ± 4	-17 ± 12	-3 ± 5	ND
9	30	5 (3-6)	-1.4 ± 1.0	25 ± 14	-24 ± 15	-1 ± 3	-11 ± 9
10	46	8 (4-9)	-1.5 ± 0.7	29 ± 16	-28 ± 17	-1 ± 4	-12 ± 9
11	21	8 (5-9)	-1.6 ± 1.1	27 ± 17	-22 ± 16	-1 ± 3	-18 ± 16
12	8	ND	- 0.9 ± 1.1	24 ± 18	-24 ± 18	-0.3 ± 1	-16 ± 12
13	28	4 (1-8)	-1.7 ± 0.9	17 ± 13	-15 ± 14	-2 ± 4	-10 ± 8
14	6	ND	-1.7 ± 0.9	23 ± 15	-22 ± 17	-1 ± 2	-13 ± 12
15	4	ND	-1.5 ± 1.0	21 ± 9	-22 ± 9.0	0.3 ± 1	-7 ± 6
16	15	9 (8-10)	-1.3 ± 1.0	26 ± 16	-26 ± 17	1 ± 2	-14 ± 8
17	23	4 (3-7)	-1.2 ± 1.3	25 ± 13	-25 ± 11	-2 ± 3	-15 ± 13
18	10	3 (1-7)	-1.0 ± 1.4	44 ± 13	-43 ± 13	-1 ± 2	-25 ± 9
19	18	7 (2-9)	-1.5 ± 0.8	27 ± 16	-28 ± 15	2 ± 2	-15 ± 5
20	4	ND	ND	25 ± 10	-24 ± 5	-1 ± 1	ND
21	6	ND	-1.0 ± 0.8	23 ± 11	-22 ± 10	0.4 ± 2	-14 ± 2
22	7	4 (2-7)	-1.7 ± 0.7	14 ± 24	-12 ± 24	-2 ± 2	-13 ± 7
23	10	8 (6-9)	-1.2 ± 0.9	31 ± 16	-31 ± 17	-0.1 ± 2	-12 ± 4
24	26	ND	-1.3 ± 1.5	29 ± 17	-31 ± 16	-0.3 ± 2	-26 ± 21
25	2	ND	-1.8± 0.1	ND	ND	ND	ND
26	12	ND	-0.9 ± 1.1	20 ± 29	-20 ± 28	0.2 ± 2	-20 ± 19
27	7	ND	-1.2 ± 0.6	18 ± 20	-12 ± 22	-1 ± 1	-7 ± 5
28	1	ND	-1.6	30	-30	0	-28
29	8	ND	-1.3 ± 0.2	12 ± 11	-15 ± 8	3 ± 4	-15
30	7	ND	-1.4 ± 0.7	29 ± 14	-29 ± 14	-0.1 ± 2	-20 ± 11

GMI: glucose management indicator; IMD: index of multiple deprivation; ND: no data reported; TAR: time above range (>180 mg/dL); TBR: time below range (<70 mg/dL); TIR: time in range (70-180 mg/dL)

Data are mean ± SD unless stated otherwise

§ Number of participants at each center included in analysis

Supplement 3. Total number of reported hospital admissions, paramedic callouts and severe hypoglycemia events (not resulting in admission or paramedic callout) at baseline and follow-up in the study cohort including those who discontinued closed-loop therapy (N=435)

Admission		Baseline	Follow-up	p-value
	Hyperglycemia/Ketoacidosis	32	17	0.33
	Severe Hypoglycemia	4	3	0.99
	Other (Diabetes related) ‡	6	1	0.16
	Other (Any)	31	19	0.55
	All admissions	73	40	0.24
Paramedic callouts*	Hyperglycemia/Ketoacidosis	7	0	0.06
	Hypoglycemia	12	5	0.26
	Other (Diabetes related) ‡	3	0	0.18
	Other (Any)	9	1	0.10
	All paramedic callouts	31	6	0.008
Severe hypoglycemia (not resulting in paramedic callouts or hospital admission)§		37	26	0.77

*Callouts not resulting in hospital admission

‡For example, admission due to diabetic foot disease, gastroparesis, insulin overdose

§Using the standard definition of requiring third-party assistance to treat
